# Supplementary material for: Effects of sustained inflation pressure during neonatal cardiopulmonary resuscitation of asphyxiated piglets
Source: PLoS One. 2020 Jun 23;15(6):e0228693. doi: 10.1371/journal.pone.0228693 (PMC7310834; doi:10.1371/journal.pone.0228693)
Supplement: Attachment — Submitted filename: Response to Reviewers_final.docx [file pone.0228693.s001.docx]

**Response to Reviewers**

We would like to thank the editors and reviewers for their thoughtful comments. We have used them to improve the presentation of our manuscript.

**Academic Editor**

1. Please ensure that your manuscript meets PLOS ONE's style requirements, including those for file naming. The PLOS ONE style templates can be found at

<http://www.journals.plos.org/plosone/s/file?id=wjVg/PLOSOne_formatting_sample_main_body.pdf> and <http://www.journals.plos.org/plosone/s/file?id=ba62/PLOSOne_formatting_sample_title_authors_affiliations.pdf>

**Response: We have revised the manuscript by referring to the PLOS ONE style template.**

2. We ask that you please clarify in your methods section, whether the piglets were fully anesthetized during the 4hr recovery period. Please state whether any of the piglets died while not under anesthesia.

**Response: We have edited the** method section: “Throughout the entire experimental period” to indicate that animal was fully anesthetized during the 4h recovery period as well. We don’t have any data on whether any of piglets will die without anesthesia.

3. We noticed you have some minor occurrence of overlapping text with a previous publication,  <https://doi.org/10.1016/j.resuscitation.2018.06.013>, which needs to be addressed.

In your revision ensure you cite all your sources (including your own works), and quote or rephrase any duplicated text outside the methods section. Further consideration is dependent on these concerns being addressed.

**Response: This has been edited**

**Reviewer #1**

1. Abstract, first sentence: This is a bold statement by the authors. The authors' claim that SI during CC reduces time to ROSC is supported by their previous observations in a piglet model. However, Vali et al did not show a difference in ROSC success or time to ROSC when comparing 3:1 compression-to-ventilation (C:V) to CC+SI in a asphyxiated cardiac arrest lamb model (PMID: 28661972). Even in their current study only 3/8 piglets achieved ROSC in the CC+SI PIP 30 cm H_2_O group. In addition, in a pilot study comparing CC+SI to 3:1 C:V, 2/5 in the SI group died at <28d compared to 0/4 in the 3:1 C:V, which is a concerning trend. There is lack of evidence to support this statement and the sentence should be revised.

**Response: This has been edited.**

2. Methodology: authors claim that the endotracheal tube was clamped until asystole, which they define as "no heart rate audible during auscultation and zero blood flow in the carotid artery." However, in Figure 2, carotid blood flow is clearly not zero. The measured heart rate also does not reach zero. Based on these values, the piglets are not in asystolic cardiac arrest as there is cardiac activity resulting in cardiac output (as demonstrated by left carotid blood flow). The authors need to state that their model is one of severe bradycardia and not asystole.

**Response: To prevent bias for overall outcome, total cardiac arrest was determined by auscultation with a standard stethoscope by a single investigator (GMS), who was blinded to HR displayed by ECG and CBF. About 42% (10 out of 24) piglets still had low CBF (0 with IQR 0-9 mL); whereas the median HR was 13 (8~15)(with 38% [9 out of 24] showed pulseless electrical activity). There was a positive correlation between HR and CBF at cardiac arrest (r^2^=0.53, p=0.008). However, there were no correlations between HR and ROSC time (r^2^=0.35, p=0.098) or CBF and ROSC time (r^2^=0.18, p=0.393). The definition of asystole has been changed in the method section accordingly.**

3. Introduction, first paragraph, 3rd sentence: the referenced study (ref 9), which was published in 2007 collected data spanning the years 1991 to 2004 and is not recent. The authors should revise their sentence to more accurately reflect the period of the referenced paper.

**Response: This ha s been revised: A systemic review of newborns born between 1991 and 2004 who underwent prolonged chest compressions without signs of life at 10 minutes following birth noted 83% mortality, with 94% of survivors suffering death or severe disability [9].**

4. Figure 2: are any values significantly different compared to baseline? The hatch (#) designation does not appear on the provided the figures.

**Response: Figure 2 has been revised to add on statistical values as compared with its own baseline. Please note that the symbol designation has been changed to minimize numbers of symbol used for clarity.**

**Reviewer #2**

1. My major concern with this conclusion is that while NRP recommends intubation during CC most providers are giving mask PPV during the initial bradycardia. if a sustain inflation could restore circulation during chest compressions it would be far more pragmatic to have a non-intubated model (or at least a comparator). I doubt providers are considering an SI breath with a PIP of 30 in humans. Most of the work will have to be done in animals since a human trial would be so difficult. I suggest the authors add a third arm to determine if the delivery of these breaths could be different with a face mask compared to an intubated animal.

**Response: Thank you for this excellent suggestion: Unfortunately, our animal ethics committee has so far not allowed this approach, but given your comment we will resubmit an ethics application to compare intubation versus face mask during CC+SI.**
